# Supplementary figures and images for: Investigation of dual antiplatelet therapy after coronary stenting in patients with chronic kidney disease
Source: PLoS One. 2021 Aug 4;16(8):e0255645. doi: 10.1371/journal.pone.0255645 (PMC8336855; doi:10.1371/journal.pone.0255645)

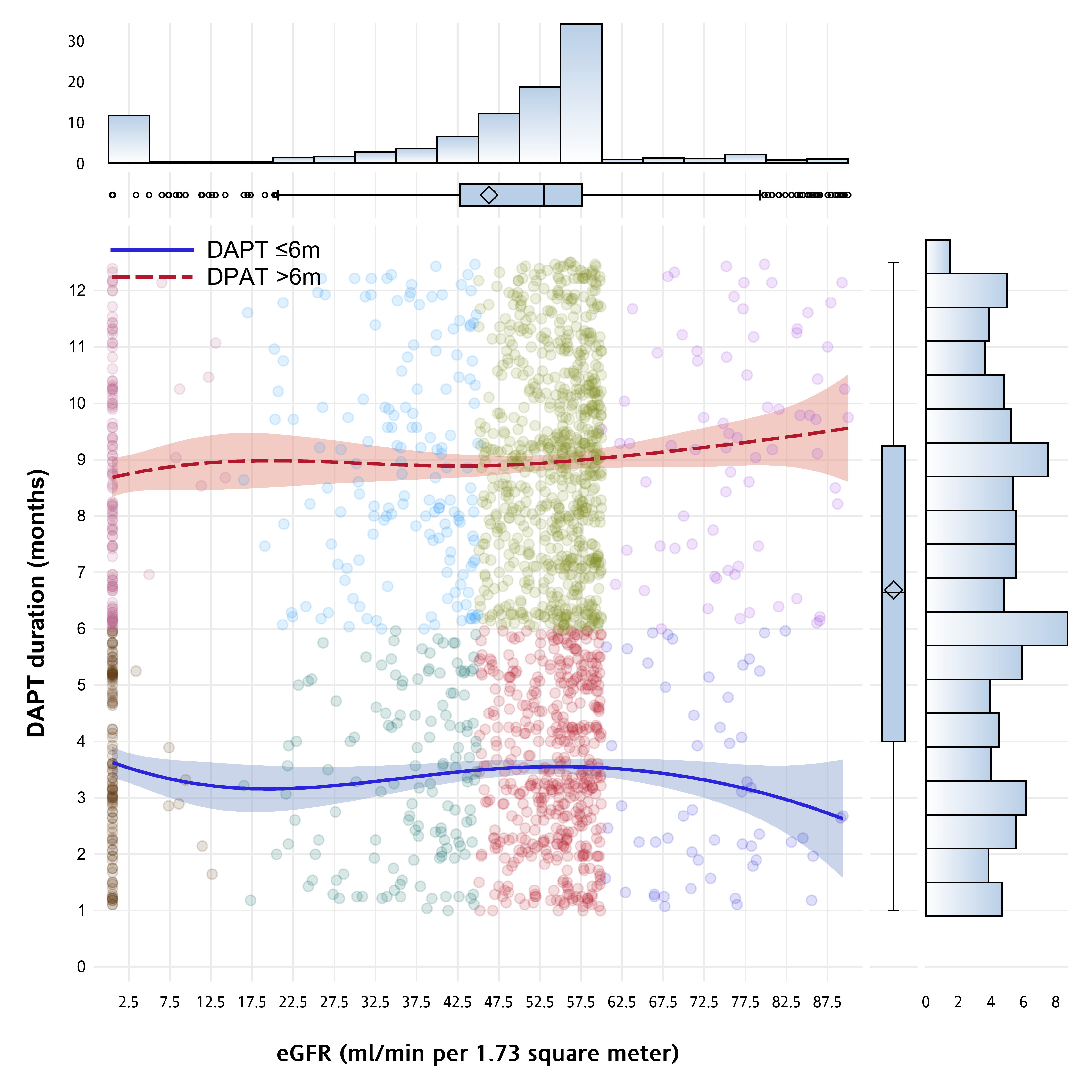

Supplement: S1 Fig — (TIF) [file pone.0255645.s001.tif]

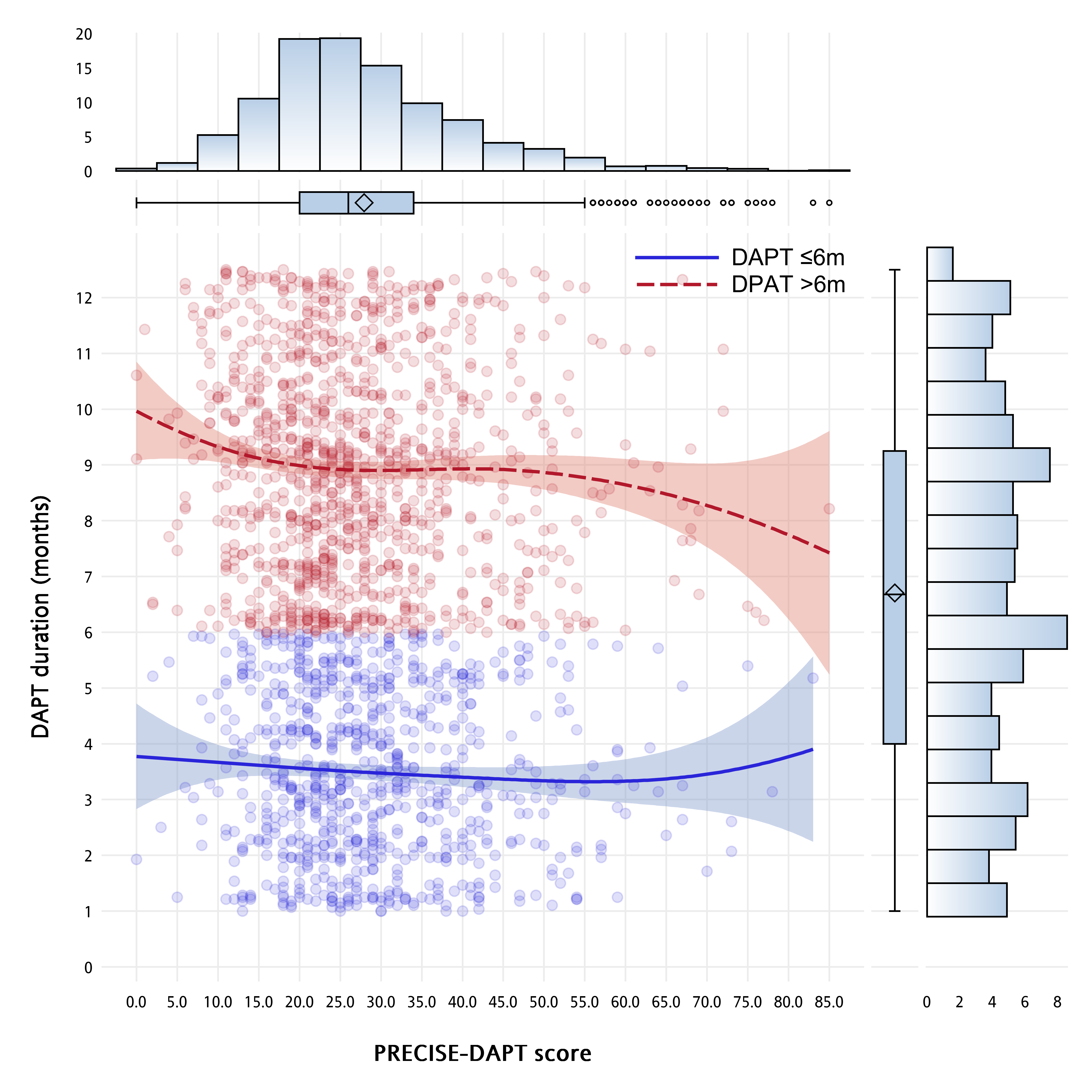

Supplement: S2 Fig — (TIF) [file pone.0255645.s002.tif]
