# Supplementary material for: Investigation of dual antiplatelet therapy after coronary stenting in patients with chronic kidney disease
Source: PLoS One. 2021 Aug 4;16(8):e0255645. doi: 10.1371/journal.pone.0255645 (PMC8336855; doi:10.1371/journal.pone.0255645)
Supplement: S1 Table — (DOCX) [file pone.0255645.s003.docx]

**S1 Table: Diagnosis codes and medication codes.**

| **Variable** | **ICD-9-CM / ATC code** | **ICD-10** |
| --- | --- | --- |
| MACE |  |  |
| Ischemic stroke | 433, 434, 436 | I61 |
| Myocardial infarction | 410 | I21 |
| Cardiovascular death | 433, 434, 436, 410, 414.00, 414.01, 414.02, 414.03, 414.04, 414.05, 441.8 | I00-I99 |
| TIMI bleeding |  |  |
| Gastrointestinal | 456.0, 456.20, 530.7, 530.82, 531.0, 531.2, 531.4, 531.6, 532.0, 532.2, 532.4, 532.6, 533.0, 533.2, 533.4, 533.6, 534.0, 534.2, 534.4, 534.6, 535.01, 535.11, 535.21, 535.31, 535.41, 535.51, 535.61, 537.83, 562.02, 562.03, 562.12, 562.13, 569.3, 569.85, 578.0, 578.1, 578.9 | K92.0, K92.1, I85.0, I98.20, I98.3, K22.10, K22.12, K22.14, K22.16, K25.0, K25.2, K25.4, K25.6, K26.0, K26.2, K26.4, K26.6, K27.0, K27.2, K27.4, K27.6, K28.0, K28.2, K28.4, K28.6, K29.0, K63.80, K31.80, K55.20, K62.5, K92.2 |
| Other non-critical site | 287.8, 287.9, 599.7, 596.7, 770.3, 784.7, 784.8, 786.3 | N02.0, N02.1, N02.2, N02.3, N02.4, N02.5, N02.6, N02.7, N02.8, N02.9, K66.1, N93.8, N93.9, N95.0, R04.1, R04.2, R04.8, R04.9, R31.0, R31.1, R31.8, D68.3, H35.6, H43.1 H45.0, M25.0 |
| Intracranial | 430, 431, 432, 432.0, 432.1, 432.9 | I60, I601-I609, I61, I611-I616, I618, I619, I62, I620, I621, I629 |
| Other critical site | 362.81, 363.61, 363.62, 376.32, 379.23, 423.0, 459.0, 568.81, 719.1 | R58 |
| AMI | 410 |  |
| STEMI | 410.[0-6,8-9][0-2] |  |
| NSTEMI | 410 excluded 410.[0-6,8-9][0-2] |  |
| HTN | 401-405 |  |
| DM | 250 |  |
| Hyperlipidemia | 272 |  |
| PAOD | 440.0, 440.2, 440.3, 440.8, 440.9, 443, 444.0, 444.22, 444.8, 447.8, 447.9 |  |
| CHF | 428, 402.11, 402.91, 404.11, 404.13, 404.91, 404.93 |  |
| Stroke | 430-437 |  |
| AF | 427.3 |  |
| Cancer | 140-208 |  |
| Aspirin | B01AC06, N02BA01 |  |
| Clopidogrel | B01AC04 |  |
| Ticagrelor | B01AC24 |  |
| ACEI | C09AA |  |
| ARB | C09CA, C09DB01, C09DB02 |  |
| Beta-blocker | C07A |  |
| PPI | A02BC |  |
| Statins | C10AA, C10B |  |

Abbreviations: ICD-9-CM, International Classification of Diseases, 9th Revision, Clinical Modification; ICD-10, International Statistical Classification of Diseases and Related Health Problems, 10th Revision; ATC, Anatomical and Therapeutic Classification; MACE, Major Adverse Cardiac Event; TIMI, thrombolysis in myocardial infarction; AMI, acute myocardial infarction; STEMI, ST-elevation myocardial infarction; NSTEMI, non-ST-elevation myocardial infarction; HTN, Hypertension; DM, Diabetes mellitus; PAOD, peripheral arterial occlusion disease; CHF, congestive heart failure; AF, Atrial fibrillation; ACEI, angiotensin converting enzyme inhibitor; ARB, angiotensin receptor blocker; PPI, proton pump inhibitor.
