# Supplementary material for: Investigation of dual antiplatelet therapy after coronary stenting in patients with chronic kidney disease
Source: PLoS One. 2021 Aug 4;16(8):e0255645. doi: 10.1371/journal.pone.0255645 (PMC8336855; doi:10.1371/journal.pone.0255645)
Supplement: Attachment — Submitted filename: Response to Reviewers_20210629.docx [file pone.0255645.s004.docx]

Response to reviewers

Subject: “Investigation of dual antiplatelet therapy after coronary stenting in patients with chronic kidney disease”

Dear Editor-in-Chief of PLOS ONE,

The authors are more than honored to receive this decision mail, and would like to express our gratitude for your re-consideration of our revised manuscript. The reviewers are all experts in this field, and their opinions are very helpful for improving our manuscript. Most importantly, we have carefully reviewed the suggestions and our responses are as follows. We also revise the manuscript by using track changes where the portion needs to be changed. We have read through all the reviewers’ suggestions very carefully and made the necessary revisions based upon your comments as detailed below in a point-by-point format.

We hope that the revised manuscript will meet the expectations of both the reviewers and yourself, and await the decision concerning publication of our manuscript in PLOS ONE.

On behalf of all authors,

Chih-Wei Chen, MD

Department of Internal Medicine and Cardiovascular Research Center

110 Taipei Medical University Hospital, Taipei, Taiwan

Tel: +886-2-27372181 ext.3577

E-mail: [ians.yahoo@gmail.com](mailto:ians.yahoo@gmail.com)

Tzu-Hao Chang, Professor

Graduate Institute of Biomedical Informatics

Research Center for Artificial Intelligence in Medicine, Taipei Medical University Professional Master Program in Artificial Intelligence in Medicine

Taipei Medical University, Taipei, Taiwan

Tel: +886-2-66382736 ext.1508

E-mail: [kevinchang@tmu.edu.tw](mailto:kevinchang@tmu.edu.tw)

Journal Requirements

Comment 1

Please review your reference list to ensure that it is complete and correct. If you have cited papers that have been retracted, please include the rationale for doing so in the manuscript text, or remove these references and replace them with relevant current references. Any changes to the reference list should be mentioned in the rebuttal letter that accompanies your revised manuscript. If you need to cite a retracted article, indicate the article’s retracted status in the References list and also include a citation and full reference for the retraction notice.

**Response: Thank you for the kind reminder. We checked the references and confirmed no retracted paper in the reference list.**

Comment 2

Please ensure that your manuscript meets PLOS ONE's style requirements, including those for file naming.

**Response: We follow your suggestion to revise the manuscript to meet PLOS ONE’s style requirements. We change level 1 heading to 18pt font and level 2 heading to 16pt font. And we also change our figure and table citation format to meet the style of PLOS ONE.**

Comment 3

Thank you for including your methods section:  "This study has been reviewed by the Institutional Review Board of TMU (TMU-JIRB No. N201707020). The need for informed consent was waived owing to the use of anonymized data."   
1. Please add this information to your ethics statement in the online submission form.

2. Please amend your current ethics statement to include the full name of the ethics committee/institutional review board(s) that approved your specific study. Once you have amended this/these statement(s) in the Methods section of the manuscript, please add the same text to the “Ethics Statement” field of the submission form (via “Edit Submission”).

**Response: Thank you for the comment. We have added this ethics statement in the online submission form and we also revised our description in method section (page 7, line 2, method section) to “This study has been reviewed by the Institutional Review Board of Taipei Medical University (TMU-JIRB No. N201707020). The need for informed consent was waived owing to the use of anonymized data.”**

Comment 4

Thank you for providing the date(s) when patient medical information was initially recorded. Please also include the date(s) on which your research team accessed the databases/records to obtain the retrospective data used in your study.

**Response: Thank you for the comment. Our research team applied to access Taipei Medical University Institutional and Clinical Database after this study was approved by the Institutional Review Board of Taipei Medical University. Our first analysis of this database was on February 06, 2020.**

Reviewers’ comments

Reviewer #1:

Methods: Confusing ? as this is a retrospective chart review and the authors have mentioned they enrolled the patients, please make it clearer what is study type and data collected? Fix both in abstract and methods. Define the clear aim in manuscript in methods.

**Response: Thank you for the valuable comment. Our study is a retrospective cohort study use anonymized data from Taipei Medical University (TMU) Institutional and Clinical Database. This database included 3 million patients’ electronic medical record form TMU Hospital, Wan Fang Hospital, and Shuang Ho Hospital respectively. The aim of our study is to compare the effectiveness and safety between long-term and short-term dual anti-platelet therapy in patients with chronic kidney disease after coronary stenting.**

We revised the description in abstract (page 3, line 9, abstract section) to “This retrospective cohort study analyze data from the Taipei Medical University (TMU) Institutional and Clinical Database, which include anonymized electronic health data of 3 million patients that visited TMU Hospital, Wan Fang Hospital, and Shuang Ho Hospital. We also added the aim of our study in methods section (page 8, line 13) “The aim of this study is to compare the effectiveness and safety between long-term and short-term DAPT in patients with CKD risk after coronary stenting.”

Reviewer #2:

This is an interesting article about DAPT use in CKD patients. The authors demonstrated that longer (>6 months) vs. shorter (< 6 months) have similar MACE and TIMI bleeding risk. This article is in line with the recent published data about the safety of shorter DAPT. CKD patients are prone to bleeding but also have higher rates of ACS and ischemic events, which makes this paper interesting for readers

**Response: Thank you for the comment. CKD patients are considered to have higher ischemia and bleeding risk compare to general populations. This is the reason why we want to conduct this study to compare the effectiveness and safety between long-term and short-term DAPT in these patients in real world setting.**
